# Natural Compounds of *Salvia* L. Genus and Molecular Mechanism of Their Biological Activity

**DOI:** 10.3390/biomedicines11123151

**Published:** 2023-11-27

**Authors:** Gaziza Zhumaliyeva, Aizhan Zhussupova, Galiya E. Zhusupova, Ewelina Błońska-Sikora, Antonella Cerreto, Nargul Omirbekova, Zhazira Zhunusbayeva, Nadezhda Gemejiyeva, Madina Ramazanova, Małgorzata Wrzosek, Samir A. Ross

**Affiliations:** 1 Department of Molecular Biology and Genetics, Al-Farabi Kazakh National University, Al-Farabi Ave. 71, Almaty 050040, Kazakhstan; zh.gaziza95@gmail.com (G.Z.); nargul.omirbekova@kaznu.kz (N.O.); zhazira.zhunusbayeva@kaznu.kz (Z.Z.); 2 Department of Chemistry and Technology of Organic Substances, Natural Compounds and Polymers, NPJSC Al-Farabi Kazakh National University, Al-Farabi Ave. 71, Almaty 050040, Kazakhstan; zhusupova@gmail.com (G.E.Z.); 3 Department of Pharmaceutical Sciences, Collegium Medicum, Jan Kochanowski University, 25-406 Kielce, Poland; eblonska@ujk.edu.pl (E.B.-S.); 4 Department of Chemistry and Technology of Drugs, Sapienza University of Rome, 00185 Rome, Italy; antonella.cerreto@uniroma1.it (A.C.); 5 Institute of Botany and Phytointroduction, 36D/1 Timiryazev Str., Almaty 050040, Kazakhstan; ngemed58@mail.ru (N.G.); r.madin.c@mail.ru (M.R.); 6 Department of Biochemistry and Pharmacogenomics, Faculty of Pharmacy and Laboratory of Biochemistry and Clinical Chemistry at the Preclinical Research Center, Medical University of Warsaw, 02-097 Warsaw, Poland; 7 School of Pharmacy, University of Mississippi, P.O. Box 1848, University, MS 38677, USA; sross@olemiss.edu (S.A.R.); 8 School of Pharmacy, S.D. Asfendiyarov Kazakh National Medical University, Almaty 050000, Kazakhstan

**Keywords:** sage species, Kazakhstan, physiologically active components

## Abstract

The study of medicinal plants is important, as they are the natural reserve of potent biologically active compounds. With wide use in traditional medicine and the inclusion of several species (as parts and as a whole plant) in pharmacopeia, species from the genus *Salvia* L. are known for the broad spectrum of their biological activities. Studies suggest that these plants possess antioxidant, anti-inflammatory, antinociceptive, anticancer, antimicrobial, antidiabetic, antiangiogenic, hepatoprotective, cognitive and memory-enhancing effects. Phenolic acids, terpenoids and flavonoids are important phytochemicals, which are primarily responsible for the medicinal activity of *Salvia* L. This review collects and summarizes currently available data on the pharmacological properties of sage, outlining its principal physiologically active components, and it explores the molecular mechanism of their biological activity. Particular attention was given to the species commonly found in Kazakhstan, especially to *Salvia trautvetteri* Regel, which is native to this country.

## 1. Introduction

A large amount of literature data suggests that different natural and herbal compounds demonstrate promising results as immunomodulatory agents with minimal side effects [1,2,3,4]. Plants as a whole or their parts have been long used in order to improve the immune status of the body [5,6,7,8,9].

Plants contain an evolutionarily developed complex of biologically active compounds, including native proteins, essential oils, trace elements, vitamins and other substances that enter into complex interactions. Therefore, despite the pronounced pharmacological effect of the “active ingredients” of phytopreparations, their overall therapeutic effect consists of the sum of multiple effects of all plant components on the organs and functional systems of the human body. In addition, many plants contain chemicals, which may act on various pathological processes. Thus, one medicinal plant may replace several synthetic agents and in therapy against disorders of various origin [10,11,12,13,14].

Being rich in terpenes, representatives of *Salvia* L. species have been widely used in the traditional medicine of different countries [15,16,17,18].

The current review is focused on the active ingredients of sage plants, which may be valuable to provide prospective options for the long-term treatment of many diseases.

## 2. General Characteristics of the Genus

Comprising 900 species, the *Salvia* genus is the largest in the Lamiaceae family. The main centers of species diversity are Central and South America (500) and the Mediterranean with Central Asia (250) and East Asia (more than 90 species) [7,19]. The diversity of *Salvia* in Central Asia is understudied: the first detailed summary of the Central Asian variations is presented by S.N. Kudryashov (1937) [20], indicating 19 species of sage.

A.M. Makhmedov (1984, 1987) [21] gives a significantly expanded composition of species, 34 species, including 20 endemics. According to the latest floristic checklists by Khassanov (2015) and Li et al. (2020) [22,23], there are 41 native *Salvia* species in the flora of Central Asia (24 of which are endemic), and 13 species are found on the territory of Kazakhstan: *S. abrotanoides* (Kar.) Sytsma, *S. aethiopis* L., *S. deserta* Schangin., *S. dumetorum* Andrz., *S. macrosiphon* Boiss., *S. karelinii* J.B. Walker, *S. korolkowii* Regel & Schmalh., *S. nemorosa* L., *S. spinosa* L., *S. sclarea* L., *S. virgata* Jacq., and *S. verticillata* L., with *S. trautvetteri* Regel being endemic to the flora of Kazakhstan [23].

Sage has long been used as a flavoring agent in food, aromatics and cosmetics. Some species are known from a wide variety of applications in traditional medicine [24] and might be recommended as a safe and effective remedy for treatment of many diseases. All species of sage are valuable to humans, displaying a wide range of pharmacological activity, including antitumor, anti-inflammatory, antinociceptive, antioxidant, antimicrobial, antimutagenic, anti-dementia, hypoglycemic and hypolipidemic effects. Each species is individual in terms of the content of essential oil (EO), phenolic acids, flavonoids and terpenic compounds. Characteristics of some of them are presented below.

*S. deserta* Schang. (desert sage) is a herbaceous perennial plant with 35–90 cm long stems. The stems are several, rarely single, longer than the inflorescence, branched in the upper part, and pubescent from the very base. The leaves are small, basal and dry up early. Inflorescences are simple, with 1–2(3) pairs of lateral branches. The corolla is dark purple and short pubescent on the outside. The fruit is dark brown, triangular–spherical nuts. The plant blooms in June–August and bears fruit in August–September. In general, it can be met in the mountains of Central Asia, Caucasus, Western Siberia and China [25,26]. The aerial part of *S. deserta* contains 0.02% of EO and the seeds contain 23% fatty acids [25]. The aerial part of *S. deserta* contains triterpenoids (ursane, oleanane and lupine derivatives) [26], while diterpenes (royleanone, ferruginol, taxodione) and caffeic acid derivatives (rosmarinic acid, lithospermic acid B and the steroid daucosterol) are mostly found in the roots [27]. Chemical constituents of *S. deserta* are scarcely presented in the literature data with studies focused on its antimicrobial, antileishmanial and antithrombotic activities [25,28].

*S. sclarea* L. (clary or musk sage) is a perennial plant with a straight stem covered with fine hairs, and it has bright inflorescences. The leaves can reach 30–35 cm in length and up to 20 cm in width. They are egg-shaped with fine wrinkles, pointed at the ends, and located on elongated petioles. The bush can bloom all summer. As a weed, it grows on any soil: rocky, clay, sandy. Growing mainly on arable land and mountain slopes, it reaches a height of 1 m or slightly higher under favorable conditions. Sage blooms in June–July, and its fruits ripen in June–August [25]. Clary sage is rich in EO, containing 45–87% linalyl acetate, 0.3–3.2% geranyl acetate, and small amounts of neryl acetate and bornyl acetate. The content of monoterpene alcohols is significant, including linalool, geraniol, nerol, a minimum of citronellol, and terpineol. Monoterpenes (α- and β-pinene, camphene, β-myrcene, cis- and transocymene, limonene), sesquiterpenes and their derivatives (germacrene D, β-caryophyllene, α-copaene, β-element, β-bourbonene, δ-kadinen, β-eudesmol, α-humulene and α-bisabolol) and oxides (1,8-cineolkaryophyllene oxide) are present in insignificant amounts. Clary sage EO has antioxidant, antibacterial, antifungal, anti-inflammatory and antiviral activity as well as high wound-healing ability. Sage oil is used in the treatment of burns and long-lasting ulcers, stomatitis and gingivitis [15,16].

*S. stepposa* Des.-Shost. (steppe sage) is a perennial plant with stems 35–60 cm high. The stem in the lower part is glabrous or pubescent with sparse short hairs. The corolla is blue–purple, 13–18 mm long, the basal rosette of the leaves is not pronounced, and the inflorescence is sparse, with 4–6 flower heads. The calyx is pubescent with glandular hairs. It blooms in June–July, and the fruits ripen in June–August [25]. In traditional medicine, dried sage leaves are used (usually infusion) as an astringent, disinfectant, anti-inflammatory and aromatic means for rinsing the mouth and throat, with advantages against gum inflammation, stomatitis, bleeding from the gums, and diseases of the teeth and throat [15].

*S. officinalis* L. (common or medicinal sage) is a perennial herbaceous plant or semi-shrub 20–80 cm tall. The stems are numerous, branched, tetrahedral, densely deciduous, and woody at the base, and the young ones are gray from numerous covering hairs. The leaves are opposite, gray–green, and densely pubescent. Its homeland is the Mediterranean, but it is cultivated all over the world [25]. Diverse uses in traditional medicine are recorded [29].

*S. nemorosa* L. (woodland sage, blue sage or wild sage) is a herbaceous perennial plant native to a wide area of central Europe and western Asia [25]. The flowers contain 0.02–0.04% EO, the composition of which has not been studied. The seeds contain up to 19% of the fat-drying oil [30]. Di- and triterpenes (like nemorone, nemorosin, horminone, 7-acetylhorminone, and salvinemorol), megastigmane glycosides (salvionosides A, B and C), pachystazone, salvipisone, α-amyrin, ursolic and oleanolic acids, stigmast-7-en-3-one, 24-methylenecycloartanol, stigmast-4-en-3-one, β-sitosterol, and stigmast-7-enol, as well as flavone aglycones apigenin, luteolin, eupatilin and salvigenin, have been isolated from its aerial parts [31].

*S. verticillata* L. (lilac sage) contains a variety of polyphenols, volatile oils, and diterpenoids. Monoterpenes display protective effects against pentylenetetrazole-, picrotoxin- and N-methyl-D-aspartate-induced convulsions. Flavonoids of *S. verticillata* are known to bind to the γ-aminobutyric acid (GABA)-type A-benzodiazepine site and may enhance the receptor sensitivity for endogenous GABA, which is a desired effect in the treatment of epilepsy [32]. Extracts of aerial parts showed cytotoxic activity on human cancer cell lines along with antimicrobial activity [33].

*S. aethiopis* (Mediterranean sage or African sage) is a perennial plant native to Eurasia. Morphologically, it is 50–120 cm high, with a thick, ribbed, shaggy pubescent, simple stem, and strongly branching only in the inflorescence; the inflorescence is strongly branched, with numerous, 6–10-flowered whorls; the corolla is white and medium-sized. It blooms in May–June and bears fruit in July–August. Diterpenes and sesterpenes were noted as major chemical constituents of *S. aethiopis* [34,35,36].

### Kazakhstani Species of Sage

A large number of Lamiaceae species (e.g., *Lamium album*, *Nepeta cataria*, *Melissa officinalis*, *Origanum vulgare*) can be found almost everywhere in Kazakhstan with the widest representation in the mountain areas of Western Tyan Shan, Tarbagatai, Kyrgyz and Ile Alatau, Karatau, which is followed by Balkash and the western, central and northern parts of Kazakhstan. At the same time, endemic species, such as *Salvia trautvetteri* and *Thymus altaicus*, grow on limited territories (in Karatau; Altai, Saur and Soongari Alatau, correspondingly). More than 10 species of *Salvia* (*S.*) grow in Kazakhstan (Appendix A, Table A1), with *S. deserta* growing on steppes, forest edges, river banks, weed, near housing and in crops, *S. sclarea* common in the foothill and foothill steppes of southern Kazakhstan (Karatau, Talas Alatau) and Dzhambul regions, *S. stepposa* widely distributed in central (Turgay, western Melkosopochnik), northern (Tobolsk-Ishim, Irtysh, Kokchetav districts), Western (Caspian, Aktobe districts, Mugodzhary) and eastern (Semipalatinsk borovoi, Altai, eastern Melkosopochnik) Kazakhstan, *S. nemorosa* widely distributed in all regions of Kazakhstan, but most abundantly in the south, within the southern Kazakhstan, Dzhambul and Almaty regions, and *S. aethiopis* distributed in the Almaty, western Kazakhstan, Zhambyl and Turkestan regions [25,35,36,37,38,39,40,41,42,43,44].

According to the study of Y.K. Levaya and G.A. Atazhanova, it was found that about 86 sage-containing drugs are available on the Kazakhstani market, of which most are presented in solid dosage forms [45]. Sadly, only 13% of those drugs are produced locally [46]. In Kazakhstan, the volume of the locally produced medications covers only 17–20% of the country’s pharmaceutical market, out of which the prevailing number are generics [47]. At the same time, in Kazakhstan, the medicinal flora is represented by 1406 species belonging to 134 families of higher flowering plants, out of which at least 50 species are pharmacopeial [42,43].

## 3. Main Biologically Active Compounds of Sage

A large number of biologically active compounds, including carbohydrates, alkaloids, fatty acids, glycosidic derivatives, phenolic compounds, terpenes/terpenoids, polyacetylenes, steroids, and waxes are found in sage. For instance, EO obtained from the aerial parts of *S. officinalis* contains more than 120 components with a domination of borneol, caryophyllene, elemene, humulene, ledene, pinene, camphor, 1,8-cineole and thujone. The latter three were evidenced as potent antimutagenic constituents [48].

Thujone is also a main constituent of several medicinal herbs with antidiabetic and antinociceptive properties [49]. In an in vitro study, thujone ameliorated insulin resistance in skeletal muscle induced by palmitate. Thujone is reported to act as an antagonist on GABA and 5-HT3 receptors in the brain [50].

More than 160 polyphenols have been identified in the *Salvia* species, some of which are unique to the genus. Caffeic acid occurs mainly in the dimer form as rosmarinic acid in *Lamiaceae* family. It is a building block for a variety of plant metabolites from monomers to oligomers, and a large number of polyphenolic compounds are constructed from it via diverse condensation reactions. Trimeric and tetrameric forms are more interesting from a therapeutic point of view, since they have significant biological activities. For instance, salvianolic acid B, a caffeic acid tetramer, is the main phenolic compound with high biological activity in *Salvia miltiorrhiza* (danshen) and the related species [51].

Rosmarinic acid, rutin, ellagic acid, chlorogenic acid, and quercetin are the most abundant flavonoids in *S. officinalis* infusion extract. The most common carbohydrates described in it are arabinose, galactose, glucose, mannose, xylose, uronic acids and rhamnose [52]. Some of this and other compounds are shown in Table 1 (see below).

## 4. Pharmacological Properties

*Salvia* species not only show antibacterial, antioxidant, free radical absorbing, anti-inflammatory and antitumor activities but also look promising in developing novel natural medicines to prevent, control and treat many diseases, such as obesity, cerebral ischemia, diabetes, cancer and Alzheimer’s disease. Sage tea and tea infusions of *S. officinalis* are effective to improve the diabetic condition [35]. *Salvia* EO and its constituents act by various pathways and mechanisms, including cell cycle arrest, apoptosis, antimetastatic and antiangiogenic activities, DNA repair modulation, and increasing the amount of reactive oxygen species (ROS) and reactive nitrogen species [99,100].

### 4.1. The Impact on Immune Cells and Cytokines Production

Immunomodulators of plant origin are valuable for the treatment of various immunologic and inflammatory diseases. The use of herbal medicines in immune-based therapy is increasingly growing. It is well known that macrophages play a crucial role in tissue inflammation response for eliminating invading pathogens. Carnosol modulated the expression of cytokine and chemokine genes and pro-inflammatory interleukins (IL), which regulate acute and chronic inflammatory processes in macrophages and chondrocytes. It also inhibited the IL-1β induced nuclear translocation of NF-κBp65, upregulated Keap1/Nrf2 pathway and positively affected the dephosphorylation of the FoxO3a. At the same time carnosol reduced ROS production [100,101].

Treatment with combined dry extract of *S. officinalis* and bitter apple in the mouse model of chronic dextran sulfate sodium colitis was characterized by a decreased histopathological score indicating tissue healing by down-regulating different stages of the inflammatory process. The recruitment of macrophages was induced and the expression of inflammatory markers was significantly suppressed [102].

Ten tanshinones isolated from *S. miltiorrhiza* significantly inhibited the expression of TNF-α and IL-8 in lipopolysaccharide-stimulated macrophages [103].

It has been shown that the extracts of *S. officinalis* exhibited a remarkable anti-inflammatory potential inhibiting the expression of TNF-α, IL-6, and IL-1β. Treatment with extracts of *S. officinalis* is able to decreases pro-inflammatory effects mediated by the breast cancer cells conditioned media [104].

The effect of sage extracts on neurophils, macrophages, T- and B-lymphocytes numerically or functionally can be explained in the light of active constituents that act either separately or synergistically in enhancing the responsiveness of these immune cells directly or indirectly [105].

*S. plebeia* significantly increased phagocytic activities, NO production and pro-inflammatory cytokines TNF-α and IL-1β in murine macrophage cell line Raw264.7 cells as well as NK cell activities and cytokine IL-12 in splenocytes [106].

Dietary supplementation of *S. officinalis* extract enhanced splenocytes of fish primary cell culture immune response to lipopolysaccharide (LPS) by the upregulation of genes involved in humoral immunity (LYS, IgM), pro- (TNF-A, IL-1β) and anti-inflammatory (TGF-β1, IL-10) cytokines, the leucocyte cell surface marker CD4, and antioxidant stress enzymes (Mn-SOD, Cat) [107].

Extract from *S. officinalis* stimulated transcriptional innate and adaptive immune responses in gut, through the modulation of processes involved in the activation, differentiation and selection of T-cell. In addition, it increased the number of intestinal goblet cells and modified the glycosylation properties of lectins from mucins [108].

Supplementation of sage EO in feed of experimental chickens led to increase in in vitro lymphocytes proliferation to mitogenic stimulus, which suggested higher immune defense abilities of the body [109].

*S. officinalis* extract effects on VCAM-1 and IL-17 signaling, demonstrated across multiple cellular models, could support modulation of neurotransmitter metabolism, reduce neuroinflammation and help to support cognitive function [110]. Another extract of this plant disposed protection against bacterial LPS-induced inflammation in human dermal fibroblast BJ cells, inhibiting the increase in IL-33 and TNF-α levels, stimulated NFκB expression and diminished the secretion of transcription factor STAT3 involved in the perpetuation of inflammation and aggravation of skin lesions [111].

It was found that thujone stimulated cell-mediated immunological response in normal and tumor bearing Balb/c mice. It enhanced the total white blood cell count, bone marrow cellularity, number of α-esterase positive cells, number of plaque forming cells in spleen and circulating antibody titer in Balb/c mice, proliferation of splenocytes and thymocytes, both in the presence and absence of specific mitogens [35].

Thujone reduced the production of pro-inflammatory cytokines, such as TNF-a, IL-1β, IL-6, and granulocytemonocyte colony-stimulating factor [35].

#### The Impact on Blood Cells

A study demonstrated that EtOAc soluble fractions of *S. deserta* root had significant antithrombotic effects (strongly impeding ADP-induced platelet aggregation rate, FeCl_3_-induced rat common carotid artery thrombus weight and thrombus area ratio), which could be attributed to platelet adhesion and aggregation inhibition as well as anticoagulant activities [26]. Moreover, they significantly decreased TXB2, vWF and PAI-1 levels, while they increased 6-keto-PGF1α and t-PA levels in plasma. Thus, the ratio of TXB2/6-keto-PGF1α was significantly decreased, while the ratio of t-PA/PAI-1 was significantly increased, underlining the anti-platelet aggregation effects of *S. deserta* extract in the rabbit and rat models. In addition, enhanced protein C and antithrombin-III, important physiological anticoagulants, indicated coagulation inactivation effects [26].

Blood analysis showed that broilers fed with different levels of sage extracts had a decreased level of plasma total cholesterol, triglycerides and low-density lipoprotein (LDL) with an increased level of high-density lipoprotein (HDL) [112].

The methanolic extract of *S. officinalis* showed anti-inflammatory activity, since it inhibited the recruitment of leukocytes from circulating blood to the lesion site [108].

### 4.2. Anti-Inflammatory Activity

Sage hydroalcoholic extract exerted a topical anti-inflammatory effect by significantly inhibiting croton oil-induced ear edema [113].

The EO of *S. officinalis* significantly inhibited NO production stimulated by LPS in mouse macrophages, which demonstrates its strong anti-inflammatory potential [114]. On the other hand, *S. officinalis* had useful effects on LPS-induced inflammation and oxidative stress in rats elevating superoxide dismutase, catalase, glutathione peroxidase. Increased levels of NO, NF-kβ and TNF-α were observed [74].

It is suggested that the beneficial effect of *S. officinalis*, rosmarinic and caffeic acids on sciatic nerve regeneration could be attributed to its biological activities like antioxidative, anti-inflammatory and neuroprotective effects [115].

Sage EO demonstrated anti-inflammatory activity significantly inhibiting leukocyte chemotaxis induced by casein and reducing leukocytes migration to spermatic fascia after inflammatory stimulus [54].

The EO of *S. officinalis* inhibited the 5-LOX (IC50 = 36.15 ± 1.27 mg/L) enzyme, which can possibly be explained by the presence of 1,8-cineole (22.22%), myrcene (0.96%), β-caryophyllene (0.27%), α-pinene (1.71%), β-pinene (1.29%), camphene (4.88%) and borneol (2.64%), which were previously shown to be involved in the treatment of certain inflammations and effectively inhibit the named enzyme [116,117].

Tanshinones and salvianolic acids in *S. miltiorrhiza* have been shown to have an anti-inflammatory effect by influencing cytokine production as well as the activity of inducible NO synthase. They also inhibited HIF-1α and NF-kβ [118,119].

In our experiments, extracts of *S. deserta* and *S. sclarea* obtained by conventional and ultrasonic-assisted extraction significantly reduced the production of pro-inflammatory cytokine TNF-α in both unstimulated and LPS-stimulated peritoneal macrophages. At the same time, it had a strong stimulatory effect on the release of IL-1β in previously unstimulated macrophages remarkably in comparison to control [120]. On the contrary, when LPS-stimulated macrophages were treated with the same sage extracts, the level of IL-1β secretion in macrophages was remarkably inhibited [119]. The data obtained by us comply with those of other scientists, who demonstrated that several groups of plant secondary metabolites (phenolic compounds, terpenes, polysaccharides) impact the functional activity of macrophages [120]. For instance, *S. miltiorrhiza* extract is reported to inhibit the production of NO, PGE2, IL-6, IL-1β, and TNF-α, and chemokines expression, RANTES, CX3CL1, and CX3CR1, as well as inflammatory mediators like TLR-4 and 11β-HSD1 in LPS-stimulated RAW 264.7 macrophages [121]. On the other hand, polysaccharides obtained from *Salicornia herbacea* stimulate intact murine macrophages to produce high levels of cytokines TNF-α, IL-1β and NO [122]. Other researchers demonstrated that pretreatment with *S. miltiorrhiza* polysaccharides led to a significant reduction in TNF-α, IL-6 and IL-1β in LPS-stimulated macrophages [123].

### 4.3. Antioxidant Activity

Oxidative stress is associated with the etiology of a large number of diseases, including diabetes, chronic inflammation, and atherosclerosis as well as many mental health and neurological disorders [124].

A number of papers have reported that sage and its components display significant antioxidant activity. As such, it has been noted that terpenoids α-pinene, 1,8-cineole and camphor present in the EO of *S. lavandulifolia* are acting as cytoprotective and antioxidant compounds by inhibiting LPO, inducing Nrf2 nuclear translocation and scavenging ROS [78,86]. The complex of biologically active compounds of *S. miltiorrhiza* inhibited the production of ROS, increasing the activities of catalase, manganese superoxide dismutase, glutathione peroxidase and coupled endothelial NO synthase [125].

The treatment of H_2_O_2_-induced human colon carcinoma Caco-2 and human cervical cancer HeLa cells with aqueous extracts of *S. officinalis* and *S. fruticosa*, and their two major phenolic constituents, namely, rosmarinic acid and luteolin-7-O-glucoside, for 24 h resulted in a protective effect from oxidative DNA damage. Notably, it was reported that luteolin-7-O-glucoside was able to increase the rate of rejoining of DNA strand breaks. Preliminary treatment with sage extracts from *S. hydrangea*, *S. multicalis*, *S. macilenta*, *S. lachnocalyx*, *S. sclarea* and *S. xanthocheila* facilitated a neuroprotective effect against oxidative stress with a significant increase in cell survival [126].

The antioxidant activity of salvianolic acids Y and B was demonstrated on PC12 cells [127,128].

In addition to antioxidant properties, the administration of *S. officinalis* decoction to gastric and small bowel injured rats abolished acute ethanolic-induced oxidative stress in the gastric and duodenal mucosa, which is an effect possibly related to a high content of flavonoids and phenolic acids [120]. *S. elegans* decoction has shown antioxidant effects, scavenging free radicals DPPH, NO and O_2_, reducing Fe^3+^, being the most effective inhibitor of α-glucosidase. It also exhibited good inhibitory capacity against xanthine oxidase activity, which was possibly related to the high content of glycosidic forms of flavones in it, such as apigenin, scutellarein and luteolin [129].

The crude methanol extract of *S. verticillata* and its ethyl acetate fraction showed strong antioxidant activities in DPPH and β-carotene/linoleic acid tests. Further fractionation and purification of the ethyl acetate fraction using chromatography showed the presence of chrysoeriol known for high antioxidant capacity [130].

Doğan et al. demonstrated the radical scavenging capacities of five *Salvia* species (*Salvia macrochlamys* Boiss., *Salvia kronenburgii* Rech.f., *Salvia euphratica* Montbret. ex Aucher var. euphratica, *Salvia huberi* Hedge and *Salvia kurdica* Benth) in the range of 84.1 ± 4.5 to 96.8 ± 0.1% [131].

The antioxidant activity of 11 abietane diterpenoids, 3 apianane terpenoids, 1 anthraquinone, and 8 flavonoids isolated from the leaves of *S. officinalis* was evaluated. Abietane diterpenoids, such as carnosol, isorosmanol, rosmanol, epirosmanol, carnosic acid and galdosol, demonstrated remarkably strong activity almost equivalent to that of α-tocopherol [132].

The antioxidant capacity of the EO obtained by hydro-distillation from the dried aerial parts of *S. officinalis* reached 33.61% and 84.50% by DPPH and ABTS analyses, respectively, which are values specific to samples rich in 1,8-cineole [133].

The EO of wild *S. officinalis* leaves effectively scavenged the DPPH radical dot free radicals with an IC_50_ value of about 8.31 ± 0.55 mg/L. This important antioxidant activity can possibly be related with a high content of 1,8-cineole (22.22%) as well as some minor components, such as α-pinene (1.71%), terpinen-4-ol (0.8%) or linalool (0.28%), which are considered to be among the most potent free radical scavengers [116].

Luca et al. studied the chemical profile and biological activities of ten Moldavian sage species from ex situ crop cultures. LC-HRMS/MS metabolites profiling allowed isolating 19 diterpenes, 15 phenolic and organic acids, 18 flavonoids, 5 sesterpenes, and 2 triterpenes. According to the results of DPPH, ABTS, and FRAP assays, *S. officinalis* was shown to be one of the most potent radical scavenging and metal-reducing agents (CE50 values of 25.33, 8.13, and 21.01 μg/mL, respectively), which was followed by *S. verticillata*, *S. sclarea*, *S. kopetdaghensis*, *S. aethiopis*, and *S. tesquicola*. Pearson correlation analysis revealed strong correlations with the presence of rosmarinic acid, luteolin-O-glucuronide, and hydroxybenzoic acid [134].

### 4.4. Hepatoprotective Activity

Pretreatment with the EO of *S. officinalis* led to a significant reduction in the levels of AST, ALT, ALP, creatinine, cholesterol and triglycerides as well as malondialdehyde (MDA). There was a notable increase in the viability of the cells and total antioxidant capacity. The presence of chemotypic components (i.e., α-pinene, camphene, β-pinene, myrcene, 1,8-cineole, α-thujone, and camphor) was associated with high activity against induced liver damages in active cancer cell lines (MCF-7, HepG-2, and HeLA), according to the in vitro hepatoprotective effect of sage [130].

The application of EO from *S. officinalis* led to normalization of the lipoprotein metabolism and lipid disturbance in rat vanadium-induced liver injury, which might be attributed to the modulation of detoxification enzymes [135].

Animals pretreated with water extracts from *S. officinalis* (containing a mixture of salvianolic acid A and B, rosmarinic acid and phenolic glycosides) failed to show necrosis of the liver after azathioprine administration. In addition, *S. officinalis* dropped the elevated levels of ALT and AST in the serum. The azathioprine-induced oxidative stress was relieved through MDA reduction and counteracting the inhibitory effect of azathioprine on glutathione, catalase and superoxide dismutase activity [136].

*S. miltiorrhiza* and other *Salvia* species have been shown to inhibit acute liver injury induced by D-galactosamine, protect against CCl4-induced acute and chronic liver injury and fibrosis, and protect against hepatic fibrosis and apoptosis induced by biliary obstruction in rats [136].

### 4.5. Cognitive Effects

Sage contains a diverse range of active compounds that may affect cognitive skills, including memory, attention, learning and dementia [27]. Alzheimer’s disease starts with subtle alterations of hippocampal synaptic efficacy prior to neuronal degeneration and deposits of fibrillar Aβ protein and NFTs in the brain, which are the pathological hallmarks of the disease. The activation of inflammatory mediators and reduction in BDNF are the potential mechanisms for Alzheimer’s disease [130].

Rosmarinic acid has shown neuroprotective, antioxidant and anti-apoptotic effects against the toxicity of Aβ (beta-amyloid plaques) in nerve cells, providing a pharmacological basis for the traditional use of sage in treatment of Alzheimer’s disease [27]. It increases BDNF and plays an important role in supporting the survival of existing neurons, encouraging the growth and differentiation of new neurons and synapses [137].

The administration of *S. miltiorrhiza* (the main constituent of Chinese medicine Danshen) to mice prevented the spatial memory impairment and neuronal damage induced by the injection of Aβ_25–35_. The mechanism of these neuroprotective effects may relate to elevated levels of choline acetyltransferase (ChAT) and receptor of activated protein kinase C1 (RACK1) and restored the balance between cytokines (IL-6 and TNF-α) and neurotrophins (BDNF) in the brain [138].

*S. officinalis* and *S. lavandulifolia* improve cognitive performance through the modulation of acetylcholine, which plays a central role in several aspects of cognitive function and behavior, including attention, learning, memory and motivation. This is due, specifically, to the inhibition of AChE, which is an enzyme that catalyzes the breakdown of acetylcholine. Using a combination of *S. officinalis* leaf extract mostly characterized for polyphenol content and *S. lavandulaefolia* EO with predominant terpenoids, a study observed improvements in memory and effects to Y-maze and Morris water maze markers on rodents in vivo [139,140,141]. This specific combination was also shown to be effective for the working memory accuracy of performance cognitive domains in healthy humans [142].

*S. miltiorrhiza* extracts and single constituents have been shown to have positive effects in central nervous system neuronal injury and degeneration in several animal models by various biological mechanisms [143].

The flavonoid luteolin was found to be highly active in inducing the synthesis and secretion of neurotrophic factors, such as nerve growth factor, glial-derived neurotrophic factor and BDNF in cultured rat astrocytes. Carnosic acid, carnosol, tanshinones and quercetin have been provided to enhance the production of nerve growth factor, which is a neurotrophin essential for the growth, maintenance and survival of neurons [144].

The EO of *S. officinalis* leaves exhibited significant inhibitory activity toward the AChE enzyme (IC50 = 38.71 ± 2.09 mg/L), which may be related to rather high 1,8-cineole (22.22%), α-pinene (1.71%) and β-pinene (1.29%) content [116].

### 4.6. Antiangiogenic Activity

The antiangiogenic activity of *S. officinalis* extract and its hexane, ethyl acetate, n-butanol and aqueous fractions was studied using (human umbilical vein endothelial cells) HUVEC capillary tube formation and rat aorta models in a three-dimensional collagen matrix. The results demonstrated that the hexane fraction of *S. officinalis* extract showed strong antiangiogenic activity both in vitro and ex vivo: this effect can be attributed to the inhibition of endothelial cell induced migration and proliferation [145].

*S. officinalis* showed strong antiangiogenic effects and significantly inhibited the blood vessels’ growth in chicken embryo chorioallantoic membrane, which is associated with the expression of VEGF, matrix metalloproteinases, and the epidermal growth factor receptor as well as the inhibition of NF-kβ, PI3-K/Akt, ERK1/2 signaling pathways [146].

Direct antiangiogenic properties of ethanolic crude extracts of *S. dominica*, *S. syriaca*, *S. triloba* and *S. hormium* leaves were studied using a rat aortic ring assay, HUVEC, cytotoxicity assay, migration assay and CAM assay. *S. triloba* showed very promising antiangiogenic activities, inhibiting human umbilical endothelial cell proliferation, migration and VEGF expression as well as HIF-1 mRNA in the breast cancer cells under both normoxic and hypoxic conditions. HIF-1α is one of the major transcription factors involved in angiogenesis. Hence, the downregulation of HIF-1α is associated with an antitumor effect; it may suggest a possible use of the latter in a specific therapy for cancer [147].

### 4.7. Anticancer Activity

It has been reported that sibiriquinones A and B, cryptotanshinone and dihydrotanshinone from *S. miltiorrhiza* inhibited luciferase expression induced by hypoxia on AGS cells (a human gastric cancer cell line) and on Hep3B cells (a human hepatocarcinoma cell line). Sibiriquinone A and dihydrotanshinone suppressed the HIF-1α accumulation, and sibiriquinone A also inhibited mRNA expression of VEGF under hypoxia. The anticancer activity of tanshinones is at least in part related to HIF-1 accumulation [148].

The extract of *S. plebeia* and its active component cosmosiin effectively blocked the molecular interaction between programmed cell death ligands 1 and 2 as well as enhanced T-cell-mediated antitumor activity [149].

Sage extracts and their components—specifically, chrysin, taxodione and ferruginol—demonstrated broad-spectrum anticancer activities, including regulation of the initiation, progression and survival of many cancer cells, making them good candidates for cancer therapy [150].

EO of *S. officinalis* and its main compounds α-thujone, 1,8-cineole and camphor demonstrated a cytotoxic effect on LNCaP cells (prostate carcinoma), MCF7 cells (breast carcinoma) and HeLa cells (cervical carcinoma). This effect is likely associated with the ability of EO to penetrate through the cell wall and cytoplasmic membrane [99].

Anticancer effects of tanshinones were studied on highly invasive human lung adenocarcinoma cell line CL1-5. Tanshinone I of *S. miltiorrhiza* reduced the transcriptional activity of IL-8, an angiogenic factor involved in cancer metastasis, by attenuating the DNA-binding activity of activator protein-1 and NF-kβ in macrophage-conditioned medium-stimulated CL1-5 cells in vitro [151].

In this context, tanshinone IIA may suppress proliferation in A549, a human non-small cell lung cancer cell line, decrease the expression of VEGF and VEGF receptor 2 (VEGFR2) in tumor cells, indirectly inhibiting the downstream PI3K/AKT signaling pathway, downregulating the expression of the anti-apoptosis gene, thereby inhibiting the growth of tumor cells as a consequence of cell apoptosis. Tanshinone IIA positively affected the progression of cell cycle from the G1 to S stage [152].

A-humulene and trans-caryophyllene extracted from the EO of *S. officinalis* displayed cytotoxic activity on tumor cell lines (HCT-116, MCF-7, RAW264.7) [84].

Sclareol isolated from *S. sclarea* was found to inhibit proliferation and induce the apoptosis of several cancer cell lines [153,154].

Evidence indicated that diterpenoids, aethiopinone and salvipisone from *S. sclarea* exhibit cytotoxic activity toward HL-60 and NALM-6 leukemia cells. These compounds act through the induction of caspase-3, one of the major proteases involved in apoptosis, in a concentration-dependent manner after short time exposure [155].

13-epimanoyl oxide and the n-hexane fraction of *S. macrosiphon* revealed significant cytotoxicity against two different human breast cancer cell lines (MCF-7 and MDA-MB-231) and a lung cancer cell line (A-549) [156].

### 4.8. Antimicrobial Activity

*Salvia* species rich in EO, such as *S. officinalis* with volatile monoterpenoids as their major constituents, are reported to have an effective antibacterial aspect, which is in part due to their hydrophobicity. They are partitioned into the lipid bilayer of the cell membrane, making it more permeable and causing a leakage of vital cell contents. The loss of the differential permeability of the cytoplasmic membrane is the cause of bacteria cell death [157]. Gram-positive bacteria are more sensitive to sage EO compared to other types of bacteria [158,159].

Recent studies have shown strong broad-spectrum antimicrobial activity of EO from *S. sclarea* [159]. It has also been shown as promising for the incorporation into nanoemulsion prepared by microfluidization. Antimicrobial activity was noted against *Saccharomyces cerevisiae*, *Geotrichum candidum*, and *Pichia pastoris* (MIC: 0.5–1.0, 0.375–0.875, 1.0, and 0.5–1.0 µL/mL, respectively); 20,000 µg/mL of EO prolonged the lag phase of *Escherichia coli* (3.20~6.40 h) and inhibited *Listeria monocytogenes* [160,161,162,163].

In a reported study by Delamare et al., the EO of *S. officinalis* and *S. triloba* exhibited notable bacteriostatic and bactericidal activities against *Bacillus cereus*, *Bacillus megatherium*, *Bacillus subtilis*, *Aeromonas hydrophila*, *Aeromonas sobria* and *Klebsiella oxytoca*. Furthermore, the EO of *S. triloba* effectively inhibited the growth of *Staphylococcus aureus* [77].

The EOs of *S. officinalis* and *S. nemorosa* are characterized by a high content of oxygenated sesquiterpenes and notable antifungal activities against *Candida albicans* and *Mycrosporum canis* [77].

Such compounds were shown to be responsible for the antifungal and antibacterial activity of sage EO: α-pinene, β-pinene, borneol, and bornyl acetate [164,165].

The EO of *S. sclarea* and linalyl acetate combination (1:1) showed significant in situ protection of *Picrorhiza kurroa* rhizomes against *Aspergillus flavus* and other biodeteriorating fungal isolates due to its high phenolic content and antioxidant nature [166].

The antimicrobial testing of EO of *S. sclarea* and its components (E)-caryophyllene and meropenem showed moderate activity. *S. sclarea* EO demonstrated the best antimicrobial effects against Gram-positive bacteria *Staphylococcus aureus* (MIC50 of 1.48 and MIC90 of 1.59 µL/mL), while meropenem was found to be the most effective against Gram-negative bacteria *Yersinia enterocolitica* with an MIC50 of 0.73 and an MIC90 of 0.98 µg/mL. For (E)-caryophyllene, the highest activity was noted against the yeast *Candida krusei* with an inhibition zone of 7.67 ± 0.58 mm. Moreover, the EO of *S. sclarea* significantly inhibited the biofilm formation of *Pseudomonas fluorescens* growing on plastic and stainless-steel surfaces, affecting bacteria homeostasis [167].

The antimicrobial activity of hydrolates prepared by microwave-assisted extraction from five *Salvia* sp. against selected strains of Gram-positive bacteria (*Micrococcus luteus* DSM 1790 and *Bacillus subtilis* DSM 5552) and strains of Gram-negative bacteria (*Escherichia coli* CCM 3954 and *Enterobacter asburiae* CCM 8546) was studied. The hydrolates prepared from *S. officinalis* and *S. sclarea* demonstrated inhibitory activity on tested Gram-positive and Gram-negative bacteria, while there was only partial inhibition by *S. nemorosa*. *Enterobacter asburiae* was the only taxon with sensitivity to *S. aethiopis* and *S. divinorum* hydrolates [168].

The antibacterial activity of extracts obtained by UAE from the leaves and flowers of *S. stepposa* growing in the territory of Kazakhstan was assessed in vitro. Both types of extracts showed strong antibacterial activity against Gram-positive bacteria *Bacillus subtilis* and *Staphylococcus aureus*. A 30% UAE extract of *S. stepposa* flowers showed the most noted antibacterial effect in relation to *Staphylococcus aureus* with growth inhibition zones of 35 ± 1 mm, while a 40% UAE extract of *S. stepposa* leaves was the best in relation to *Bacillus subtilis* with growth inhibition zones of 49 ± 1 mm, and a 70% UAE extract of *S. stepposa* leaves was the best for *Escherichia coli* with growth inhibition zones of 24 ± 1 mm [169].

In a more recent study, 45 isolates of endophytic microorganisms were isolated from three species of sage, growing in Kazakhstan (*S. aethiopis, S. nemorosa, S. sclarea*), most of which were isolated from the roots (42.2%) with the least number (24.4%) isolated from the leaves of the studied plants. Two strains of endophytic actinomycetes, N.A1 and N.A4, were found to effectively suppress the growth of *Staphylococcus aureus*, *Aspergillus niger*, *Candida albicans*, and *Fusarium oxysporum* with inhibition zones ranging from 10.0 to 18.2 mm. Among the studied endophytic bacteria strains, S.B4 of *Bacillus* genus was chosen, as it effectively suppressed the growth of *Staphylococcus aureus, Escherichia coli,* and *Aspergillus niger*. The growth inhibition zones ranged from 10.3 to 14.5 mm [170].

### 4.9. Antidiabetic Activity

Herbal antioxidants can activate reduction–oxidation-sensitive transcription factors as well as cellular antioxidant and detoxification capacities. Studies showed that *Salvia* sp. plants contain numerous flavonoids like apigenin 7-glucoside, 8-di-C-glucosyl apigenin, luteolin 7-glucoside, luteolin 7-diglucoside, and phenolic acids, which possess antioxidant and anti-inflammatory activities [171].

The results demonstrated that the methanolic extract of *S. spinosa* effectively decreased oxidative stress and renal injury in streptozotocin-induced diabetic mice, reducing the level of blood urea nitrogen and serum creatinine, improving the histopathological state in the diabetic mice and preventing mitochondrial disfunction [172].

The oral administration of *S. officinalis* extract for 14 days exhibited a significant reduction in serum glucose, total cholesterol, triglycerides, urea, creatinine, uric acid, aspartate aminotransferase, and alanine aminotransferase as well as increased plasma insulin in streptozotocin-induced diabetic rats [173].

In alloxan-induced diabetic mice, the extracts of *S. officinalis* had antihyperglycemic efficacy. After 7 days of oral administration of aqueous extract, a lower level of fasting blood sugar, without toxic effects on the kidney or liver, was reported, and the hypoglycaemic effect was even better than after the administration of glibenclamide [174].

Moreover, *S. officinalis* tea, similarly to metformin, had an effect on hepatocytes in mice and Wistar rats. The authors showed the decrease in glucose concentrations and production in these animals. In addition, the inhibition of gluconeogenesis was reported. This increase in hepatocyte sensitivity to insulin in animals indicates the potential for sage tea to prevent diabetes [175].

The neuroprotective and antidiabetic properties of *S. officinalis* (most active as AChE, BChE and α-glucosidase inhibitor), *S. glutinosa* (most active as α-amylase inhibitor) and *S. transsylvanica* were also studied [176].

Also, human studies showed the potential of *S. officinalis* in treating diabetes. It was shown that glucose and cholesterol concentrations significantly decreased in patients with type 2 diabetes treated with *S. officinalis*. This improvement of the glycemic and lipid profile suggests beneficial effects of sage against insulin resistance [177,178].

## 5. Nephroprotective Properties

Decoction extract from the flowers of *S. officinalis* exerted a protective effect against ethanol-induced injury in the rat liver and kidney by plasma transaminases activity and preservation of the hepatic tissue structure [179].

A study by Ahn et al. [180] showed that the chronic oral administration of Tanshinone IIA can improve renal dysfunction related to chronic kidney disease. Moreover, tanshinone IIA has renoprotective effects against the progression of diabetic nephropathy. *S. miltiorrhiza* decreased urine protein excretion by 65% in streptozotocin-induced diabetic nephropathy [181].

The in vivo results revealed that *S. officinalis* aqueous extract (SOE) mitigated disturbances and histological alterations in the liver and kidney tissue in rats treated by CdCl_2_. The kidney sections of the rats treated with SOE revealed renal tissue with a normal tubular and glomerular structure. Moreover, SOE administration induced a loss of body weight and a notable elevation in liver and kidney indices, NO, cholesterol, triglycerides, LDL, serum cytokines, hepatic and renal malondialdehyde, mRNA expression of *Bax* gene accompanied by a significant decrease in HDL, hepatic and renal antioxidant enzymes and their mRNA gene expression [182].

The nephroprotective effect of EO from the aerial parts of *S. officinalis* seems to be related to a high content of β-caryophyllene, limonene, carvacrol, caryophyllene, borneol, α-pinene, and α-thujene. The administration of EO from this species significantly restored biochemical markers and pathological lesions on renal nephrotoxicity induced by vanadium in rats [183,184]. Hosivandi et al. studied the protective effects of *S. macrosiphon* methanolic extract on renal ischemia reperfusion, which was shown to significantly reduce the level of urea and creatinine [185].

Kim et al. discovered that the extract of *S. plebeia* inhibited the activity of xanthine oxidase, which is the main enzyme producing uric acid in the liver. In an animal model of hyperuricemia, *S. plebeia* extract reduced serum urate to the levels observed in control animals. The urate-lowering effect of *S. plebeia* extract in vivo was supported by the identification of compounds (nepetin, scutellarein, and luteolin) inhibiting xanthine oxidase enzyme activity in vitro [186]. Lithospermic acid isolated from the extract of *S. miltiorrhiza* roots had marked in vivo hypouricemic and anti-inflammatory effects on rats [187].

The study of Zhang et al. demonstrated the uricosuric and nephroprotective effects of the ethyl acetate extract of *S. miltiorrhiza* (EASM) and tanshinone IIA (Tan-IIA) on uric acid nephropathy. After treatment, decreased serum uric acid and creatinine levels were observed in experimental mice. Both EASM and Tan-IIA demonstrated inhibitory effects on uric acid nephropathy through the downregulating of cyclooxygenase-2 (COX-2) and inducible nitric oxide synthase (iNOS), relieving NOX4-mediated oxidative stress and suppressing MAPK pathways activation [188].

The EO of *S. officinalis* leaves inhibited xanthine oxidase (XOD), which catalyzes the generation of uric acid. This inhibitory activity might be associated with the presence of α-thujone, β-thujone, α-pinene, β-pinene, β-caryophyllene and myrcene [116].

A study carried out by Bahadori et al. demonstrated that *S. spinosa* was a potentially effective xanthine oxidase inhibitor (IC50 = 38.7 ± 0.5 mg/L), which may be related to the presence of caryophyllene and spathulenol [189]. Moreover, the methanolic extract of *S. spinosa* showed an XO inhibitory activity of 71.5% [190].

## 6. Conclusions and Future Perspectives

**Figure 1:**
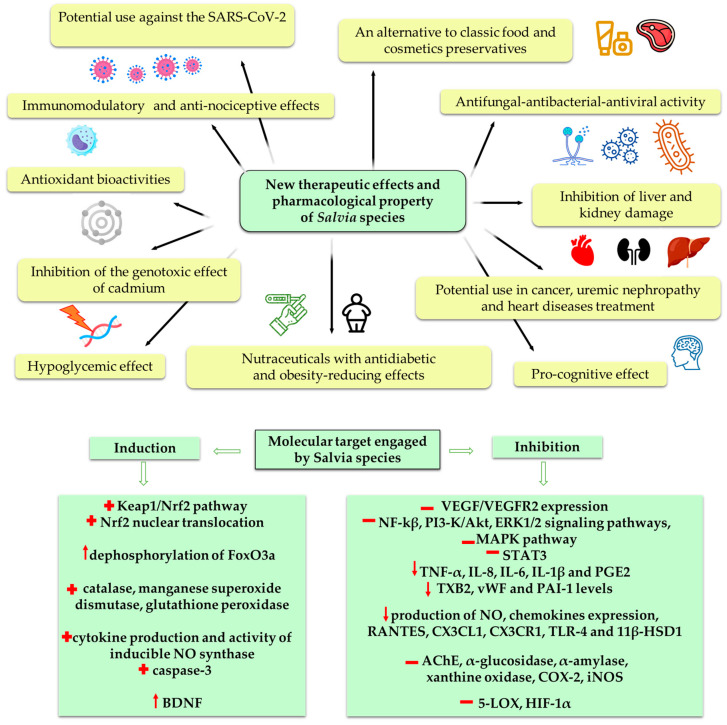
The summary of the novel therapeutic effects and pharmacological properties of *Salvia* species and the impact on molecular pathways. Possible places of inhibition (−) and activation (+) by *Salvia* species.

Studies suggest that plants of the *Salvia* genus possess antioxidant, anti-inflammatory, antinociceptive, anticancer, antimicrobial, antidiabetic, antiangiogenic, hepatoprotective, cognitive and memory-enhancing effects. These herbal medicines have been found to be very effective in the development of novel natural drugs to prevent, control, and treat many health problems. The summary of the novel therapeutic effects and pharmacological properties of *Salvia* species is presented in Figure 1. *Salvia* extracts contain a number of biologically active compounds, including phenolic acids, terpenoids, flavonoids, polysaccharides and essential oils, which have tremendous capability to maintain and/or stimulate the immune system. Sage products and biologically active compounds modulate immune responses through the stimulation and modification of lymphocytes, macrophages, and cytokine production. Extensive pharmacological and chemical experiments on *Salvia* plants provide a basis for future research on the application of medicinal plants. Much of the research about the biological activities of *Salvia* species was conducted as studies in vitro and in animal models, which we reviewed thoroughly in this paper. The molecular pathways engaged by *Salvia* species are presented in Figure 1. The diverse biological potential of *Salvia* species have been observed, including the upregulation and downregulation of different biological factors. However, the clinical interpretation of some of the studies is limited due to the unknown mechanisms behind the reported biological activities. Thus, further studies must confirm their utility in the prevention and support of the treatment of chronic diseases before their clinical implementation. Moreover, plant extracts or their phytochemicals demonstrated different biostability and bioavailability, which may profit from novel technologies for encapsulation, such as biopolymer nanoparticles, emulsions, liposomes, solid lipid or biopolymer nanoparticles. Last but not least—the administration of particular plant-derived products might substantially improve the treatment of many diseases. Nevertheless, there is still much to be achieved. Considering all advantages of using medicinal plants, further studies beyond in vitro tests are recommended. In fact, only human studies can demonstrate the clinical applicability of the in vitro findings.

## Figures and Tables

**Table 1:** Promising biologically active compounds of sage.

Compound	Structure	Plant Name	Biological Activity	References
Phenolic acids				
Caffeic acid	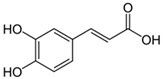	*S. sclarea*, *S. officinalis*, leaves	anti-inflammatory antioxidant, antineuropathic, neuroprotective	[53,54,55]
Rosmarinic acid	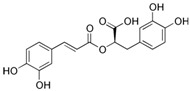	*S. sclarea*, *S. officinalis*, leaves	anti-inflammatory, antioxidant, activity on melanogenesis, antineuropathic, neuroprotective	[56,57,58,59,60,61]
Salvianolic acids	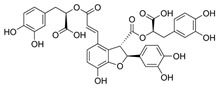	*S. miltiorrhiza*, *S. divinorum*	antioxidant, antidiabetic, hepatoprotective, neuroprotective, cardiovascular protection, antidepressant, anxiolytic	[62,63,64,65,66]
Terpenoids				
Oleanolic acid	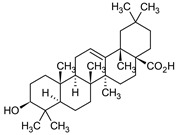	*S.officinalis*, leaves *S. sclarea*, leaves *S. virgata*, leaves	antimicrobial, anti-inflammatory, antidiabetic, anti-nociceptive	[67,68]
Ursolic acid	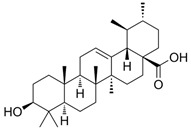	*S. officinalis*, leaves *S. sclarea*, leaves *S. virgata*, leaves	anti-inflammatory, antidiabetic, antiprotease and antimetastatic	[68,69,70,71]
1,8-cineole	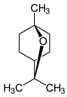	*S. officinalis*, aerial part	antifungal, antibacterial, anti-inflammatory	[70,71,72]
Borneol	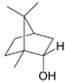	*S. officinalis*, aerial part	antifungal, antibacterial	[52,72,73]
Thujone	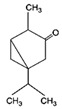	*S. officinalis*, aerial part	antifungal, antioxidant, anti-inflammatory, antiviral, antibacterial	[70,71,74,75]
Camphor	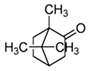	*S. officinalis*, aerial part	antioxidant, anti-inflammatory, antiviral, antibacterial, antifungal	[72,74]
Ferruginol	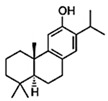	*S. deserta*, *S. hypargeia*, root, *S. miltiorrhiza*, *S. sclarea*	antimicrobial, antileishmanial, antioxidant, antielastase, anticholinesterase, antiurease, cytotoxic, antihypertensive	[44,75,76,77,78]
Linalyl acetate	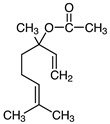	*S. officinalis*, leaves *S. sclarea*	analgesic, antiproliferative, antistaphylococcal	[79,80]
Humulene	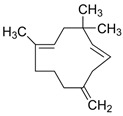	*S. sclareopsi*, *S. officinalis*	antioxidant, cytotoxic	[81,82,83,84]
Pinene	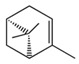	*S. lavandulifolia*, *S. sclarea*	antioxidant, antistaphylococcal	[85,86]
Carnosic acid	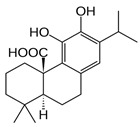	*S. officinalis*, leaves	antioxidant, antimicrobial, anticarcinogenic, anti-inflammatory, antinociceptive	[87,88,89]
Carnosol	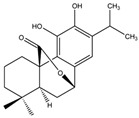	*S. officinalis*, leaves	antioxidant, antimicrobial, anticarcinogenic, anti-inflammatory, antinociceptive	[87,88,89]
Manool	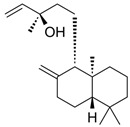	*S. officinalis*, leaves, *S. sclarea*	anti-inflammatory, antigenotoxic, anticarcinogenic, chemopreventive, cytotoxic, antibacterial	[90,91,92]
Viridiflorol	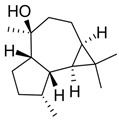	*S. dentata*, *S. sclareopsis*, *S. officinalis*	antimicrobial, antifungal, antioxidant, cytotoxic	[93,94]
Tanshinones	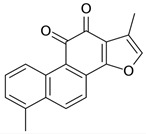	*S. miltiorrhiza*, root	antioxidant, antimicrobial, anti-tumor, cardiovascular protective, neuroprotective	[35,95,96,97]
Flavonoids				
Luteolin	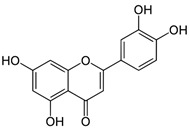	*S. sclarea*, *S. officinalis*	anti-inflammatory, antioxidant	[98]
Apigenin	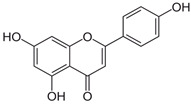	*S. sclarea*, *S. officinalis*	anti-inflammatory, antioxidant, benzodiazepine receptor activity	[5,44]
Hispidulin	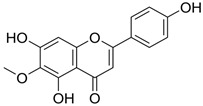	*S. plebeia*	hepatoprotective, anticancer	[16,45]
Quercetin	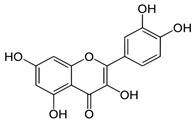	*S. officinalis*	antioxidant	[75]

## Appendix A

**Table A1:** Characteristics of the species of the genus *Salvia* L. in the flora of Kazakhstan.

Species Name (Part Used)	Distribution in Kazakhstan (Floral Area)	Ecology, Habitats	Information about the Content of Biologically Active Compounds	Therapeutic Effect	References
*Salvia aethiopis* L. (aerial part)	Chu-Ili Mountains (26), Kyrgyz Alatau (27), Karatau (28)	Steppes, meadow slopes of steppe mountains	Alkaloids, tannins, flavonoids, triterpenoids, quinones, essential and fatty oils	Antibacterial, antifungal, antihyperhidrosis	[25,37,38]
*Salvia macrosiphon* Boiss. (whole plant)	Western Tien Shan (29)	Foothills, gravelly and loess, rocky slopes, valleys and bottoms of dried rivers	Essential oil, coumarins, flavonoids, quinones, sterols, diterpenes	Antibacterial, repellent, expectorant, cardiotonic	[25,37,38]
*Salvia deserta* Schangin (aerial part)	Tobolsk—Ishim (2), Irtysh (3), Semipalatinsk forest (4), Kokchetau (5), near Caspian (6), Aktobe (7), Mugojary (7a), Turgay (9), western and eastern Melkosopochnik (10, 11), Zaisan (12), Balkhash—Alakul (18), Kyzyl—Kum (20), Turkestan (21), Altay (22), Tarbagatay (23), Dzungarian Alatau (24), Trans—Ili Kungei Alatau (25), Chu—Ili Mountains (26), Karatau (28), Western Tien Shan (29).	Steppe zone, steppe mountain slopes, forest edges, river banks, often as weeds near roads, housing, fields	Organic acids, alkaloids, tannins, flavonoids, phenolic carboxylic acids and their derivatives, quinones, essential and fatty oils, vitamins	Raw materials for the production of quinones, antibacterial	[25,37,38]
*Salvia sclarea* L. (aerial part)	Chu—Ili Mountains (26), Karatau (28), Western Tien Shan (29)	Gravelly, rocky mountain slopes, gorges and valleys	Organic acids, alkaloids, tannins, flavonoids, phenolic carboxylic acids and their derivatives, quinones, essential and fatty oils, vitamins	Anti-inflammatory, antispasmodic, tonic, diuretic, antiseptic, wound healing	[25,37,38]
*Salvia stepposa* Schost. (whole plant)	Tobolsk—Ishim (2), Irtysh (3), Semipalatinsk forest (4), Kokchetau (5), near Caspian (6), Aktobe (7), Mugojary (7a), Turgay (9), Western and Eastern Melkosopochnik (10,11), Altay (22)	Steppes, dry steppe meadows	Carbohydrate, quinones, fatty oil	Antibacterial, antifungal	[25,37,38,39]
*Salvia trautvetteri* Regel (whole plant)	Karatau (28), Talas Alatau (29). Endem	Steppe, rocky and gravelly mountain slopes	Flavonoids, quinones	Antiprotozoal, bacteriostatic	[25,37,38,39,40,41]
*Salvia virgata* Jacg.	Western Tien Shan (29)	Meadow, mountain slopes and lawns, edges of walnut and deciduous forests, often as weeds	No data available	No data available	[25]
*Salvia verticillata* L. (whole plant)	Tobolsk—Ishim (2)	Stony scree, pine forests, dry elevated places, stony-clay soil, often as a weed	Carbohydrate, terpenoids, steroids, coumarins, tannins, flavonoids, anthocyanins, quinones, essential and fatty oils	Hemostatic, astringent, wound healing	[25,37,38]
